# Comparison of Propofol and Alfaxalone as Anesthesic Drugs in Bitches Undergoing Ovariohysterectomies (Healthy Bitches and with Pyometra) and Cesarean Sections

**DOI:** 10.3390/ani14091343

**Published:** 2024-04-29

**Authors:** Raquel Rodríguez-Trujillo, Miguel Batista-Arteaga, Kseniia Iusupova, Inmaculada Rosario-Medina, Desirée Alamo-Santana

**Affiliations:** Unit of Reproduction, Universitary Institute of Biomedical Research and Health, University of Las Palmas de Gran Canaria, Transmontaña s/n, 35413 Arucas, Las Palmas, Spain; raquel.rodriguez@ulpgc.es (R.R.-T.); ksfedorchak23@gmail.com (K.I.); inmaculada.rosario@ulpgc.es (I.R.-M.); dalamosantana@gmail.com (D.A.-S.)

**Keywords:** propofol, alfaxalone, ovariohysterectomy, pyometra, cesarean section, Apgar test

## Abstract

**Simple Summary:**

This study compared the effectiveness of two anesthesia drugs, alfaxalone and propofol, for female dogs undergoing an ovariohysterectomy (*n* = 69) or cesarean sections (*n* = 28). Maternal parameters were monitored during surgery, and neonatal viability was assessed post-delivery. The results showed no significant differences in maternal parameters recorded throughout surgery between the two drugs. Propofol required more additional doses for induction, the application of this agent was always the one recommended and did not exceed its maximum dose. Neonatal mortality rates were similar, but alfaxalone was associated with better neonatal viability and required less neonatal care compared to propofol. In summary, both drugs were equally effective for ovariohysterectomies, while alfaxalone showed potential benefits for neonatal health after cesarean sections.

**Abstract:**

This study evaluated the efficacy and safety of two anesthetic agents, alfaxalone and propofol, on maternal physiological parameters (heart and respiratory rates, blood pressure, and temperature) on either ovariohysterectomies or cesarean sections in bitches. A total of 34 healthy and pyometra-affected females (classified as ASA II), were induced with IV propofol (4 mg/kg), while 35 females, both healthy and pyometra affected, were induced with IV alfaxalone (1 mg/kg). For cesarean sections, females (ASA II) were induced with propofol (*n* = 14) or alfaxalone (*n* = 14). Additionally, the neonatal viability and modified Apgar score were recorded at 5, 60, and 120 min post-delivery. There were no significant differences in the physiological parameters when comparing the use of propofol and alfaxalone in bitches undergoing ovariohysterectomies, regardless of their health status, nor when comparing cesarean sections. It was observed that bitches induced with propofol occasionally required an additional dose for maintenance of the anesthesia. Neonatal mortality rates were similar for both groups; however, alfaxalone was associated with higher neonatal viability as indicated by the Apgar scores. The findings suggest that both anesthetic protocols are effective and safe for use in canine reproductive surgeries, with no major differences in basic physiological parameters’ alteration or neonatal outcomes between the two agents.

## 1. Introduction

In small animals, surgeries of the female reproductive tract are common procedures in veterinary practice. A routine ovariohysterectomy involves the removal of both ovaries and the uterine horns [1] and is usually performed via a ventral midline approach. Pyometra is a hormone-mediated disorder in which the uterine endometrium is altered due to a secondary bacterial infection; surgical treatment of pyometra includes an ovariohysterectomy similar to the one developed in healthy bitches, although it must be completed carefully due to the greater fragility of the uterine tissues [2]. During the cesarean section, a medial ventral incision is made to gain access to the abdominal cavity, then the uterine horns are carefully exteriorized, and a small incision is made at the uterine body to access the interior of the uterus; finally, a gentle massage is applied to the uterine horns to facilitate the delivery of the neonates [3]. For all procedures, preoperative and postoperative factors must be considered, and the anesthetic protocol should be optimized to minimize neurological and cardiorespiratory depression in females.

The goal of premedication is to prepare the animal for anesthesia, which may include sedation, analgesia, or both, depending on the individual’s clinical needs. This approach can also have the benefit of sparing the amount of induction agents needed, which can be advantageous in managing the animal’s physiological response to anesthesia. Propofol and alfaxalone are two drugs commonly used for anesthetic induction in veterinary medicine. Propofol is an alkylphenol-based intravenous anesthetic agent. It is known for its rapid anesthetic induction [4] and short recovery times, and it primarily acts on the gamma-aminobutyric acid (GABA), enhancing the inhibitory effect of the neurotransmitter GABA to produce anesthesia [5]. Alfaxalone, is a synthetic neuroactive steroid that interacts with the y-aminobutyric acid (GABA) receptors of the central nervous system, producing anesthesia and muscle relaxation [6]. While both agents act on the same receptor type, their effects on physiological parameters can differ; propofol is associated with decreases in heart rate and mean arterial pressure, and also promote respiratory depression [7]. Alfaxalone, on the other hand, is reported to maintain remarkably stable cardiovascular and respiratory parameters during general anesthesia [8]. Both propofol and alfaxalone exhibit rapid distribution, metabolism, and clearance, reducing the risk of accumulation and facilitating a quick recovery from anesthesia [7].

Different studies have reported the use alfaxalone for total intravenous anesthesia in healthy bitches undergoing an ovariohysterectomy [6,9,10]. In addition, the effectiveness of propofol or alfaxalone for anesthesic induction during cesarean sections has been also evaluated [11,12,13,14] and the influence on neonatal viability was determined, showing the best results when alfaxalone was used. Furthermore, only one study [15] has assessed the effectiveness of alfaxalone for total intravenous anesthesia in bitches undergoing elective cesarean sections. Other studies have described an anesthetic protocol consisting of premedication with an opioid, followed by induction and maintenance with propofol until delivery of neonates [3]. However, no studies have been performed on cesarean sections, comparing the efficacy of propofol or alfaxalone to perform the anesthesic induction and maintenance until the neonates’ delivery. Finally, to our knowledge, no studies have been completed assessing the efficacy and safety of propofol versus alfaxalone in bitches with pyometra. 

Several studies have considered the use of an Apgar score and a short-term survival prognosis in evaluating neonatal viability [16,17,18,19,20]. Neonatal parameters assessed in APGAR are heart rate, mucous membrane color, respiratory rate, stress-induced reflexes, and neonatal activity [3,17]. Temperature, glucose, and lactate plasma concentrations are also variables to consider in neonates [21,22]. The present study was performed to assess the efficacy and reliability of alfaxalone in comparison with propofol to induce anesthesia in healthy bitches and bitches with pyometra undergoing an ovariohysterectomy. In addition, the use of propofol and alfaxalone as anesthetic induction agents for cesarean sections in bitches, and their influence on neonatal viability were also defined.

## 2. Materials and Methods

### 2.1. Animals

This study enrolled ninety-nine privately owned female dogs, categorized by breed and age, and their 124 offspring. Before undergoing surgery, all bitches were subject to a thorough physical examination, which included heart rate, glucose levels, rectal temperature, breathing frequency, and mucus color evaluation, as well as a complete blood analysis. The females were divided into two groups based on the type of surgery they received: those undergoing ovariohysterectomy, which included both healthy females and those affected by pyometra, and those undergoing cesarean sections, which were exclusively brachycephalic breeds such as the American Bully, English Bulldog, and French Bulldog. The procedures took place at the Veterinary Hospital at the University of Las Palmas de Gran Canaria over a two-year period from 2020 to 2022. All protocols were carried out in accordance with the animal welfare committee’s ethical guidelines ensuring the highest standard of care for all animals involved.

In this study, the bitches with pyometra as well as those who were pregnant and undergoing cesarean sections, were all classified as ASA II, indicating mild systemic disease with no significant functional limitations. This classification was based on the presence of mild systemic alterations, ensuring a homogeneous risk profile among the subjects for the purpose of the study. By categorizing both the pyometra bitches and the pregnant females as ASA II, we aimed to minimize the induction agent’s variability due to differences in the sensorium’s state. Therefore, it was anticipated that the amount of induction medication required would not vary widely among the subjects, reducing potential bias in the study’s design and outcomes.

### 2.2. Experimental Design

The bitches were assigned to three experimental groups according to the type of surgery performed: ovariohysterectomy in healthy bitches (OVH), ovariohysterectomy in bitches with pyometra (OVHP), and cesarean section (CS). The selection of the anesthetic protocol was random for each bitch. The anesthetist applied the randomly selected anesthetic protocol, being the only one aware of it until the surgery was completed and all the results were obtained. Those responsible for collecting anesthetic data and determining the Apgar score were unaware of the induction agent used until the end to prevent any bias in the results of each measurement.

All females were premedicated with the IM administration of methadone (0.04 mg/kg; Semfortan 10 mg/mL. CIMAvet, Spain) and, after 10–15 min, anesthesia was induced. In the OVH group (*n* = 42), venous access was established in all females prior to induction; females showing agitation during the catheter placement were excluded from the study. After premedication with methadone, which did not result in heavy sedation, 20 females were induced with IV propofol (OVHP), and 22 females with IV alfaxalone (OVHA). Similarly, in the pyometra group (*n* = 27), after securing venous catheters without incident due to the animals remaining calm and not agitated, 14 females were induced with IV propofol (OVHPP) and 13 with IV alfaxalone (OVHPA). For both groups, the induction process was smooth, with no cases of agitation affecting catheter placement or the amount of induction agent administered. Subsequently, anesthesia was maintained with sevoflurane in all cases. Regarding cesarean sections, the venous catheter was placed in a similar way to previous groups, and then they were subdivided into two groups: in group CSP (*n* = 14) induction was performed with propofol and additional boluses of propofol were administered to maintain the anesthesia until the complete delivery of puppies, after which anesthesia was maintained with sevoflurane; in group CSA (*n* = 14), induction was completed with alfaxalone, additional boluses of alfaxalone were administered to maintain the anesthesia, and once the puppies were delivered, the anesthesia was maintained with sevoflurane. Physiological parameters (heart rate, breathing frequency, blood pressure, temperature, and oxygen saturation) were recorded during surgeries in the dams of the different groups; in addition, the viability and modified Apgar score were recorded in the neonates at 5, 60, and 120 min after birth. In all surgery groups, the bitches were organized uniformly (and randomly) according to age, weight, BCS, and breed. 

### 2.3. Pre-Anesthetic Evaluation and Pre-Surgical Assessment

A clinical examination was conducted to measure the respiratory rate, heart rate, mucous membrane condition, and temperature before females were premedicated. A preanesthetic assessment was carried out, including blood tests (Procyte dx, IDEXX Laboratorios S.L, Barcelona, Spain), biochemistry analysis (Catalyst Dx, IDEXX Laboratorios, S.L, Barcelona, Spain), thorax radiography, and electrocardiogram. Regarding cesarean sections, plasmatic progesterone levels were determined before surgery and a transabdominal ultrasound (C4-1 Curved Array, ZONE Sonography^®^, Mindray Zonare Z.One PRO, Mountain View, CA, USA) was performed in the dams, and the neonatal heart rates were measured to check for fetal stress. All C-sections were scheduled between 59 and 61 days after ovulation.

Premedication included IM administration of methadone (0.04 mg/kg; Semfortan 10 mg/mL. Ecuphar, Barcelona, Spain). After the application, bitches due to have ovariohysterectomies (both healthy and those with pyometras) and cesarean sections were preoxygenated for 10 min and catheters were applied in the cephalic vein. Prior to induction, the operating room was prepared, particularly for cesarean sections.

### 2.4. Anesthetic Induction and Patient Preparation

Propofol was administered at a dose of 4 mg/kg (Propovet, 10 mg/mL, Esteve, Barcelona, Spain), while alfaxalone was used at 1 mg/kg dose (Alfaxan, 10 mg/mL, Jurox, Rutherford, Australia). During surgical procedures, including cesarean sections, after endotracheal intubation, bitches were connected to an automatic respirator. Anesthesia was initially maintained with sevoflurane post-induction for routine surgeries. However, for cesarean sections, sevoflurane was not used; instead, maintenance doses of propofol or alfaxalone were administered in boluses as needed. For all groups, the total safe dose of the induction agent was calculated based on the weight of the bitch and divided into four equal parts. If a bitch exhibited signs of pain, a positive palpebral reflex, or a superficial anesthetic plane, an additional boluse dose, equivalent to one-quarter of the total calculated dose, was given to maintain adequate anesthesia. Boluses were administered judiciously to avoid over-sedation and to ensure the safety of both the bitches and the puppies.

Continuous and automated monitoring of anesthetic parameters was conducted from induction to the end of surgery. This monitoring included heart rate, respiratory rate, blood pressure, temperature, oxygen saturation, and capnography, and was facilitated using a Ventilator GE Datex Ohmeda Anesthesia Machine (Vetmat, Bizkaia, Spain) equipped with a multi-parameter monitor that recorded these vital signs at 5-min intervals. Blood pressure was measured indirectly with an oscillometric cuff, and the cuff size was selected based on the patient’s limb circumference to ensure accuracy. To guarantee dependable readings, the equipment was calibrated prior to each surgery.

During surgery, if a female dog exhibited physiological responses indicative of nociception, such as an increase in heart rate, blood pressure, or respiratory rate, fentanyl (Fentadon 50 μg/mL, Northwich, United Kingdom) was administered intravenously. The dose of Fentanyl for rescue analgesia was 1 μg/kg. The administration was performed slowly to prevent potential respiratory depression and other opioid-related side effects. Crystalloid fluids (Ringer Lactate^®^ 3 mL/kg/h, Braun, Barcelona, Spain) were given to support blood pressure before, during, and after the procedure.

In the case of cesarean sections, once the neonates were safely removed, sevoflurane was then used for the remainder of the anesthetic maintenance.

### 2.5. Surgical Procedure

Ovariohysterectomies (in both healthy bitches and bitches with pyometra) were performed using a conventional approach. An incision was made in the skin and subcutaneous tissues to expose the linea alba, and once the abdominal cavity was opened, the uterine horns and ovaries were located. Ovarian and uterine vessels were ligated, and the reproductive tract was removed. Finally, the closure was made in layers; the abdominal wall and the subcutaneous tissue were sutured in a continuous pattern; finally, the skin was closed with an intradermal suture.

A standard surgical technique was used for the cesarean section. The uterine body and horns were carefully exposed, and a 4–10 cm incision was made in the uterine body. The neonates were swiftly extracted and transferred to the neonatal resuscitation area. After the removal of the puppies, the uterine mucous membrane and submucosa were closed using a monofilament absorbable suture (Monosyn^®^ 3/0, HR22, B. Braun Surgical SA, Rubí, Spain) in a single continuous pattern and then an inverting pattern (Cushing) was performed. Afterwards, intravenous oxytocin (1-4 IU Oxiton^®^, Ovejero, León, Spain) was administered to facilitate the expulsion of the placental membrane. Finally, the closure was performed in layers, in the same manner as the ovariohysterectomies.

### 2.6. Neonatal Resuscitation

The veterinarians responsible of the resuscitation procedure always used the same reanimation protocol without knowing the anesthetic protocol employed. Electric blankets were used to regulate their temperature. There were enough personnel in the resuscitation area to care for up to two puppies each.

The ABC protocol (airway/breathing/cardiac) was used to properly manage newborns. The airways were cleared by suctioning the mouths and noses with a bulb syringe, removing any fluids or meconium, and ensuring that the mouth and nose were devoid of fetal membrane. The newborns were rubbed vigorously with a towel, and the animal’s head was tipped downward to allow fluids to drain from the airway. The pups were not swung, thus avoiding causing excessive intracranial pressure, subdural hemorrhage, or the pass of stomach content into the airway.

Oxygenation and a heat source were available during the process. The respiratory rate was closely monitored and if the neonates were not breathing adequately, they were placed on an oxygen mask. If newborns were auscultated and found to have no heartbeat, emergency neonatal resuscitation began with heart compressions (2–3 compressions per second). If the newborn’s vitality did not improve after several minutes of resuscitation, a catheter was inserted into the jugular vein for intravenous administration of drugs. Medicaments used were as follows: naloxone (0.05–0.1 mg/kg) to reverse the effects of opioids given to the mother during premedication; heptaminol (0.1 mg/kg) to support systole and blood pressure; and epinephrine (0.2 mg/kg) to increase heart rate. ABC was stopped if neonates did not show a favorable evolution after 45 min of resuscitation.

After the puppies were stabilized, the placenta was removed, and blood was drawn to measure biochemical parameters. Also, the temperature was measured, and the puppies were weighed on the scale. Afterward, the newborns were placed in an incubator (Vetario© S40, Vetario, Weston-super-Mare, UK) at a temperature of 33–34 °C and fed before 60 min after their birth. Puppies with physical deformities such as anasarca, pronounced cleft palate, or wide fontanelle were registered. If the newborns’ welfare was incompatible due to congenital malformations, they were euthanized.

### 2.7. Apgar Test Evaluation

The Apgar test was performed to assess neonatal viability [3]. The Apgar test included five parameters: heart rate, respiratory rate, mucosal appearance, neonatal mobility, and reflex/irritability status. The heart rate was measured using a stethoscope and an ultrasound scan; the respiratory rate was determined by counting the number of spontaneous breaths per minute; the color of the mucous membrane was by direct examination; mobility was based on spontaneous movement; and reflex irritability was determined by the response to external stimuli, such as compression of a paw tip. Each parameter received a score of 0, 1, or 2 with a final score ranging from 0 to 10, as described Table 1. Puppies were classified into three categories based on their final Apgar score: normal neonates (score > 7), moderate viability neonates (score: 3–7), and critical neonates (score < 3). The Apgar test was performed at 0 (Apgar 0), 60 (Apgar 60), and 120 (Apgar 120) minutes after birth.

### 2.8. Statistical Analysis

The SPSS 10.0 software package was used to analyze the data (SPSS Inc., Chicago, IL, USA). Normality of continuous data was assessed by visual inspection of the histogram and normal quantile plot. Continuous data that are normally distributed are presented as mean ± SD; categorical variables are reported as a fraction (%). The Shapiro–Wilk test was performed to evaluate normality in data intra-operative parameters (propofol/alfaxalone doses, time intervals, secondary effects, fentanyl intraoperative, and maternal physiological parameters). Normal data (parametric) were examined with repeated measures analysis of variance (ANOVA), and a Bonferroni post-hoc test for multiple comparisons was conducted when a statistical significance was found. All data regarding Apgar evaluation and puppies’ viability (puppies born alive, neonates stillborn, and total neonatal mortality) were analyzed using the general linear models (GLMs). The lineal model included the effects of the anesthetic protocol (2 protocols), the Apgar scores (critical neonates, moderate viability neonates, and normal viability neonates), and the time of Apgar evaluation (Apgar0, Apgar60, and Apgar 120) as well as the interactions between them. Values were considered to be statistically significant when *p* < 0.05.

## 3. Results

### 3.1. Ovariohysterectomies in Healthy Bitches and Pyometra-Affected Females

The mean weight and age of healthy females (*n* = 42) were 14.34 kg and the age ranged between 8 months and 4 years. In contrast, bitches with pyometra (*n* = 27) had a mean weight of 17.12 kg and their age varied between 7 and 11 years. There were no significant differences in weight or age between the propofol and alfaxalone groups. Table 2 presents the percentage of females requiring additional doses of propofol/alfaxalone to complete the induction, observed secondary effects, and the administration of fentanyl during surgery. A higher percentage of females required additional doses to complete induction when propofol was administered (55.9%, 19/34) compared to alfaxalone (28.6%, 10/35); with no significant differences between the OVH and OVHP groups. Notably, transient tachycardia was observed as a secondary effect exclusively in the alfaxalone groups (11.4% vs. 0.0%, *p* < 0.05), which normalized after 5 min. Regarding the administration of intraoperative fentanyl, no significant differences were observed between the propofol and alfaxalone groups. 

Table 3 illustrates the mean blood pressure and heart rate in the OVH and OVHP groups. Blood pressure did not show significant differences between the groups. In females induced with alfaxalone (OVHA and OVHPA), the heart rate was significantly higher (*p* < 0.05) during the first 10 min of surgery compared to those induced with propofol (OVHP and OVHPP). However, in the second half of surgery, both groups exhibited similar values. Additionally, temperature changes throughout the surgery were minimal (range: 37.1–37.7 °C), with no significant differences observed between the groups. Heart rate and temperature were measured at 0, 30, and 60 min after surgery, and did not show significant differences between the protocols. However, a decrease in body temperature one hour after surgery in all groups was noted, but was not statistically significant (36.8, 36.9, 36.7, and 37.0 °C, mean values for OVH, OVHP, OVHA, and OVHPA, respectively). 

### 3.2. Cesarean Sections

Bitches that underwent cesarean sections had a mean weight and age of 23.5 kg and 3.4 years, respectively, with no significant differences between the propofol and alfaxalone groups. A higher percentage of females required additional doses of propofol for maintaining the anesthetic plane until the delivery of all neonates (Table 4), compared to alfaxalone (64.3% vs. 28.6%, CSP and CSA, respectively; *p* < 0.05). No differences were observed between the groups regarding the administration of fentanyl during surgery, with values ranging between 43% and 50%. Finally, secondary effects (transient tachycardia) immediately after induction did not show differences between the groups.

Table 5 presents the mean blood pressure and heart rate recorded in bitches throughout the cesarean sections. Blood pressure did not exhibit significant differences between the groups, with only a slight decrease observed in the mean values at the end of the cesarean sections. Similar to ovariohysterectomies, the heart rate showed higher values (*p* < 0.05) in the first 10 min of surgery when alfaxalone was administered, but thereafter the heart rate was similar between both protocols. Regarding temperature, mean values ranged between 37.5 and 38 °C throughout surgery. All females survived the cesarean section and experienced an appropriate postoperative recovery; bitches were consciousness within 30 min after surgery, being monitored for the next 3 h.

The incidence of neonatal mortality is detailed in Table 6. The total neonatal mortality was 8.1% (10/124), but when stillborn puppies (6/10) were excluded, the neonatal mortality after cesarean section decreased to 3.2% (4/124). No significant differences were observed between CSP and CSA, with comparable percentages of stillborn puppies and puppies that were born alive but subsequently died between 15 and 30 min after being delivered. The percentage of bitches exhibiting neonatal mortality was quite similar (35.7% vs. 28.6%, CSP, and CSA, respectively), and once the surgeries with stillborn puppies were excluded, neonatal mortality was recorded in only 10.71% (3/28) of the cesarean sections.

The mean average of the Apgar test increased slightly at each measurement in both protocols, and the difference between the Apgar 0 evaluation (7.0 ± 0.2 and 8.1 ± 0.2, CSP and CSA, respectively) and the Apgar 120 score (9.1 ± 0.2 and 9.7 ± 0.1, CSP and CSA, respectively) was markedly significant (*p* < 0.01). When both protocols were compared, it was observed that the alfaxalone protocol had higher Apgar test values (*p* < 0.05) in all three periods studied (Apgar 0: 7.0 ± 0.2 vs. 8.1 ± 0.2; Apgar 60: 8.2 ± 0.2 vs. 9.1 ± 0.1, Apgar 120: 9.1 ± 0.2 vs. 9.7± 0.1, CSP and CSA, respectively). Finally, initial viability scores (critical, moderate, and normal) for each period in both protocols are shown in Table 7. In the CSP group, immediately after birth, about 60% of the puppies were classified as normal neonates (score > 7), significantly higher (*p* < 0.01) than that recorded in moderate and critical neonates. In addition, neonates in critical condition decreased markedly at 60 and 120 min after birth, while the percentage of neonates in moderate or normal condition remained consistent. Regarding the CSA group, the percentage of neonates with normal viability was notably higher (*p* < 0.005) than the number of critical puppies in the Apgar 0 evaluation, and all neonates reached the best score at 120 min after birth. When both protocols were compared, it was observed that the percentage of normal neonates was higher in group CSA in Apgar 0, and this difference became more evident (*p* < 0.01) at 60 and 120 min after birth, with values between 95 and 100% of normal neonates in the CSA group, while in CSP group they were between 75 and 80%. Finally, in both protocols, no neonate decreased their score over time.

## 4. Discussion

Different studies have assessed the use of alfaxalone for the induction and maintenance of anesthesia in bitches. However, to our knowledge, this study describes for the first time the comparison between propofol and alfaxalone for inducing and maintaining anesthesia in all the basic reproductive surgical procedures (ovariohysterectomies, pyometras, and cesarean sections) usually performed on the bitch.

When comparing the use of propofol and alfaxalone in ovariohysterectomy procedures on healthy bitches (OVH) and those with pyometra (OVHP), no significant differences were observed in the maternal parameters assessed. During cesarean sections, propofol or alfaxalone was administered in intermittent boluses to maintain anesthesia, without the use of sevoflurane; once all neonates were delivered, sevoflurane was applied to maintain anesthesia, and no further boluses of the induction agents were given. It is important to note that this approach constituted a partial intravenous anesthesia (PIVA) rather than a total intravenous anesthesia (TIVA), due to the combination of intravenous agents with inhalant anesthesia (sevoflurane) used in different stages of the surgical procedures. Different studies have pointed out that the drug dosages may be fine-tuned based on the type of premedication used [23], and that a slower infusion rate could lead to reduced induction doses for both propofol and alfaxalone. It has been reported that alfaxalone administration can lead to an increase in heart rate as well as hypotension, especially at higher doses; whereas propofol is primarily associated with hypotension [24,25]. In our study, a transient tachycardia appeared in 9.09% of the female dogs treated with alfaxalone, but the heart rate was similar between protocols less than 10 min after anesthetic induction. During surgery, the mean arterial pressure (MAP) showed the same behavior in both protocols. A mild to moderate decrease in MAP has been described in dogs after anesthetic induction using therapeutic doses [24,25], and another study [9] reported significant changes in MAP when the suspensory ligament was pulled in female dogs if the induction and anesthetic maintenance was performed with propofol. In our study, no significant differences were observed between the protocols, probably because sevoflurane was used to maintain the anesthesia. Intraoperative pain was primarily monitored controlling the heart rate and MAP; when the heart rate increased, a microdose of fentanyl (1 microgram/kg) was administered to the bitches, with slightly higher values in the propofol group. This finding is consistent with reported results when alfaxalone is used instead of propofol for induction and/or maintenance [9].

No studies have directly compared anesthetic parameters in female dogs with pyometras using propofol or alfaxalone. In our study, we documented hemodynamic parameters in bitches with pyometra, which were classified as ASA II due to moderate analytical alterations in the red and white blood cells series. These dogs were deemed fit for surgery but, due to their condition (either being pregnant or suffering from pyometra), they were categorized as hemodynamically “unstable” in the context of the ASA classification system. Despite these moderate analytical changes, the animals were compensated and could undergo anesthesia and surgery without being considered critical. Our study demonstrated that alfaxalone and propofol can be used effectively as induction agents in these patients. In the present study, different parameters such as temperature, heart rate, and mean arterial pressure (MAP) were monitored and recorded. In both groups (alfaxalone and propofol), female dogs experienced transient tachycardia after induction, but no instances of apnea were observed. The temperature decreased in both groups during surgery, which was associated with the influence of the anesthetic agents [24]. When values were compared with those during ovariohysterectomy performed in healthy bitches, no significant differences in heart rate, temperature, or MAP were found. Some studies have reported a harder recovery when alfaxalone was used for induction and maintenance, but this may be related to the level of sedation prior to surgery [25,26,27]. However, in our study, the recovery profiles were similar across all protocols, both in healthy bitches and in bitches with pyometra. 

Several studies have found that neonates delivered by cesarean sections showed a mortality rate ranging from 4 to 15% [3,13,17,28,29,30,31]. In the present study, neonatal mortality was 8.1%, and after removing the stillborn neonates, only 3.1% (4/124) of the newborns were born alive but died within 2 h; one neonate had a severe congenital pathology (anasarca), and the other three came from cesarean sections with fetal stress. Some studies [3] reported lower neonatal mortality in cesarean sections with smaller litters (<4 puppies), but other studies have reported greater neonatal viability in caesarean sections of large litters [29]. Our study showed that neonatal mortality was more frequent in bitches with large litters and those belonging to brachycephalic breeds. This finding has been reported in other studies that show a high neonatal mortality rate (immediately after birth) associated with brachycephalic breeds [30,31]. 

In our study, when propofol was used during cesarean sections, additional doses were frequently required to maintain adequate anesthesia, with the requirement for these top-up doses being up to four times higher than that registered for bitches induced with alfaxalone. These extra doses were not part of the initial induction phase but were administered to sustain the depth of anesthesia throughout the surgical procedure. Our findings agree with different studies [12] that confirm a greater efficacy of alfaxalone in inducing a correct anesthetic plan to subsequently carry out cesarean sections, using practically similar induction doses, either propofol (4 mg/kg) or alfaxalone (2–3 mg/kg). Similarly, the physiological parameters of the mother during surgery were similar in both surgical protocols, indicating that anesthetic maintenance (until puppy delivery) can be carried out interchangeably with propofol or alfaxalone, without evident changes in temperature, heart rate, or MAP of the dam. In addition, both protocols presented the same degree of neonatal viability, similar to different studies that did not detect differences in the rate of neonatal mortality when comparing both anesthetic protocols [12,29].

The Apgar test is a useful tool to assess the neonatal viability immediately after birth or a cesarean section [18]. In our study, a lower score was measured immediately after birth (Apgar 0) and increased 120 min following delivery (Apgar 120), similar to the results described in different studies [3,31] reporting mean values between 7.5 and 8.8. The anesthetic protocol showed influence over the Apgar test, with a higher proportion of neonates in optimal condition (score 7–10) immediately after delivery when alfaxalone was used, and these results are in agreement with previous studies [11]. It has been reported that alfaxalone reduced the duration of apnea in the mother, and as a result, the neonatal score at birth was higher when compared to propofol [12], and a better viability was observed in neonates born of mothers where alfaxalone was used [11].

Based on the results of this study, the influence of the anesthetic protocol can be linked to neonatal viability. Mortality was observed in puppies showing a lower Apgar score at birth, but 100% survival was found when neonates had an Apgar score >5, independently of the anesthetic protocol. However, in the alfaxalone group, neonates presented a better Apgar test immediately after birth, confirming this trend in the following measurements. Some studies have reported that the neonates awaken from anesthesia faster and with greater vitality [11] when alfaxalone is used. This could explain that neonates born via caesarean section with propofol had a lower mean score in the first Apgar measurement when compared to the alfaxalone group. All anesthetic drugs can cross the placenta, so propofol or alfaxalone used to perform induction and anesthetic maintenance in dams may affect the fetuses. As a side note, the placental transfer of propofol is quick [32] and intravenous propofol applied until the removal of all neonates could produce the same effect (respiratory depression) on the neonates as it did on the mother. Furthermore, propofol promotes the appearance of hypotension and decreases the resistance in the circulatory system and blood flow from the heart; these effects can also be observed in neonates. However, when alfaxalone was used, maternal cardiovascular depression was minimal, resulting in a compensatory increase in heart rate. If these same effects appear in neonates, it may explain why puppies born via caesarean section with propofol had a lower Apgar score and a lower vitality immediately after birth. Regardless of the anesthetic protocol, viability improved in neonates 120 min after birth, most likely because puppies had metabolized the anesthetic drugs received through the placenta, normalizing their vital functions. Some authors have suggested that once hypoxia is removed, the respiratory and cardiovascular functions quickly return to normal, improving neonatal viability [33,34].

## 5. Conclusions

Based on the findings of this study, propofol and alfaxalone showed similar efficacy for healthy bitches and bitches with pyometra undergoing an ovariohysterectomy, with no significant differences in hemodynamic stability during surgery. Both anesthetic protocols proved to be effective for cesarean sections. Alfaxalone was associated with a higher neonatal vitality as indicated by immediate post-birth Apgar scores. However, it is important to note that this did not translate into a statistically significant difference in neonatal survival rates between the two protocols.

## Figures and Tables

**Table 1:** Apgar score protocol used in the present study.

Parameter	Score		
	0	1	2
Mucus color	Cyanotic	Pale	Pink
Heart rate (bpm)	<180 bpm	180–220 bpm	>220 bpm
Reflex irritability	Absent	Grimace	Vigorous
Mobility	Flaccid	Some flexions	Active motion
Respiratory rate (rpm)	<6 rpm	6–15 rpm	>15 rpm

**Table 2:** Percentage of additional doses of propofol/alfaxalone required to complete induction, side effects after induction, and fentanyl administration during surgery in the OVHP, OVHA, OVHPP, and OVHPA groups.

Groups	Extra Doses	Secondary Effects	Intraoperative Fentanyl
OVH_P_	55.0% ^a^ (11/20)	0.0% (0/20)	40.0% (8/20)
OVH_A_	31.8% ^b^ (7/22)	4.5% (1/22)	27.3% (6/22)
OVHP_P_	57.1% ^a^ (8/14)	0.0% (0/14)	42.8% (6/14)
OVHP_A_	23.1% ^b^ (3/13)	23.1% (3/13)	46.1% (6/13)
OVH_P_ + OVHP_P_	55.9% ^a^ (19/34)	0.0% ^a^ (0/34)	41.1% (14/34)
OVH_A_ + OVHP_A_	28.6% ^b^ (10/35)	11.4% ^b^ (4/35)	34.3% (12/35)

^ab^: Different letters in the same row and category denote significant differences (*p* < 0.05).

**Table 3:** Mean (±sd) blood pressure and heart rate during ovariohysterectomies in healthy bitches (OVH) and bitches with pyometra (OVHP).

	Blood Pressure (mm Hg)				Heart Rate (beats/min)			
Minutes	OVH_P_	OVH_A_	OVHP_P_	OVHP_A_	OVH_P_	OVH_A_	OVHP_P_	OVHP_A_
0	82.9 ± 2.1	89.0 ± 1.2	83.6 ± 3.2	85.6 ± 3.0	88.9 ± 5.1 ^a^	117.5 ± 6.2 ^b^	99.5 ± 4.1 ^a^	122.1 ± 2.9 ^b^
5	85.2 ± 2.3	80.5 ± 2.7	81.3 ± 4.2	82.2 ± 3.1	79.4 ± 4.4 ^a^	110.7 ± 4.7 ^b^	96.3 ± 3.2 ^a^	111.5 ± 4.0 ^b^
10	81.5 ± 3.1	72.5 ± 3.2	74.6 ± 2.2	75.4 ± 2.8	78.5 ± 3.0	92.6 ± 5.2	92.7 ± 6.0	102.5 ± 4.6
15	81.6 ± 2.5	75.6 ± 2.3	73.1 ± 3.4	73.2 ± 2.5	79.0 ± 3.9	85.7 ± 3.3	93.8 ± 3.5	97.5 ± 3.1
20	82.9 ± 2.1	89.0 ± 1.2	83.6 ± 3.2	85.6 ± 3.0	88.3 ± 4.2	96.6 ± 6.2	93.5 ± 3.4	90.0 ± 3.9
25	79.0 ± 3.3	86.2 ± 1.9	75.2 ± 2.9	79.6 ± 5.1	83.1 ± 4.3	81.5 ± 2.9	93.1 ± 2.1	94.1 ± 4.7
30	--	--	73.2 ± 2.3	70.6 ± 4.1	--	--	104.5 ± 5.3	106.3 ± 3.7

^ab^: Different letters in the same file and parameter (blood pressure/heart rate) denote significant differences (*p* < 0.05).

**Table 4:** Percentage of additional doses of propofol/alfaxalone, side effects after induction, and fentanyl administration during cesarean sections in experimental groups.

	Additional Doses	Secondary Effects	Intraoperative Fentanyl
CS_P_	64.3% ^a^ (9/14)	7.1% (1/14)	50.0% (7/14)
CS_A_	28.6% ^b^ (4/14)	14.2% (2/14)	42.8% (6/14)

^ab^: Different letters in the same row denote significant differences (*p* < 0.05).

**Table 5:** Mean (±sd) blood pressure and heart rate during cesarean section in bitches induced with propofol (CS_P_) or with alfaxalone (CS_A_).

	Blood Pressure (mm Hg)		Heart Rate (beats/min)	
Minutes	CS_P_	CS_A_	CS_P_	CS_A_
0	82.5 ± 3.1	88.4 ± 1.9	92.1 ± 4.1 ^a^	118.4 ± 3.9 ^b^
5	82.9 ± 4.1	80.1 ± 2.3	88.5 ± 4.5 ^a^	102.8 ± 5.1 ^b^
10	79.1 ± 5.3	81.5 ± 1.5	87.2 ± 4.9	94.6 ± 5.5
15	78.2 ± 2.5	80.8 ± 1.4	85.3 ± 3.8	91.4 ± 3.0
20	79.4 ± 2.8	81.2 ± 1.8	83.7 ± 4.0	88.3 ± 3.4
25	70.4 ± 3.7	72.5 ± 3.5	80.5 ± 3..4	84.7 ± 3.7
30	69.5 ± 5.2	70.1 ± 3.3	81.0 ± 3.7	84.5 ± 5.2
35	74.5 ± 3.3	72.7 ± 2.8	86.2 ± 4.0	89.1 ± 4.2

^ab^: Different letters in the same file and parameter (blood pressure/heart rate) denote significant differences (*p* < 0.05).

**Table 6:** Neonatal viability after cesarean sections in bitches induced with propofol (CS_P_) or with alfaxalone (CS_A_).

	CS_P_	CS_A_	Total
Neonates stillborn	4.7% (3/64)	5.0% (3/60)	4.8% (6/124)
Neonates born alive that died	3.1% (2/64)	3.3% (2/60)	3.2% (4/124)
Total neonatal mortality	7.8% (5/64)	6.7% (4/60)	8.1% (10/124)

**Table 7:** Neonatal initial viability based on the Apgar score in bitches induced with propofol (CS_P_) or with alfaxalone (CS_A_).

		Neonatal Score		
	Groups	Critical (Score < 3)	Moderate (Score: 3–7)	Normal (>7)
Apgar 0	CS_P_	9.3% ^a,1^ (5/54)	33.3% ^b^ (18/54)	57.4% ^c^ (31/54)
	CS_A_	1.7% ^a,2^ (1/58)	25.9% ^b^ (15/58)	65.5% ^c^ (38/58)
Apgar 60	CS_P_	1.8% ^a^ (1/54)	22.2% ^b,1^ (12/54)	75.9% ^c,1^ (41/54)
	CS_A_	0% ^a^ (0/58)	3.4% ^b,2^ (2/58)	96.6% ^c,2^ (56/58)
Apgar 120	CS_P_	0% ^a^ (0/54)	20.4% ^b,1^ (11/54)	79.6% ^c,1^ (43/54)
	CS_A_	0% ^a^ (0/58)	0% ^b,2^ (0/58)	100% ^c,2^ (58/58)

^abc^ Different letters in the same file and Apgar score denote significant differences (*p* < 0.01); ^1,2^ Different numbers in the same row and Apgar score denote significant differences (*p* < 0.01).

## Data Availability

Data will be available for all readers upon request.
